# Indoor Tanning and the Risk of Overall and Early-Onset Melanoma and Non-Melanoma Skin Cancer: Systematic Review and Meta-Analysis

**DOI:** 10.3390/cancers13235940

**Published:** 2021-11-25

**Authors:** Seokyung An, Kyungsik Kim, Sungji Moon, Kwang-Pil Ko, Inah Kim, Jung Eun Lee, Sue K. Park

**Affiliations:** 1 Department of Biomedical Science, Seoul National University Graduate School, Seoul 03080, Korea; Seokyung.ann@gmail.com (S.A.); kks6235@snu.ac.kr (K.K.); 2 Department of Preventive Medicine, Seoul National University College of Medicine, Seoul 03080, Korea; kajaman3@snu.ac.kr; 3 Cancer Research Institute, Seoul National University College of Medicine, Seoul 03080, Korea; 4 Interdisciplinary Program in Cancer Biology, Seoul National University College of Medicine, Seoul 03080, Korea; 5 Clinical Preventive Medicine Center, Seoul National University Bundang Hospital, Seongnam-si 13620, Korea; kpkono1@gmail.com; 6 Department of Occupational and Environmental Medicine, College of Medicine, Hanyang University, Seoul 04763, Korea; inahkim@hanyang.ac.kr; 7 Department of Food and Nutrition, Seoul National University, Seoul 08826, Korea; jungelee@snu.ac.kr; 8 Integrated Major in Innovative Medical Science, Seoul National University College of Medicine, Seoul 03080, Korea

**Keywords:** indoor tanning, melanoma, non-melanoma skin cancer, squamous cell carcinoma, basal cell carcinoma, lentigo maligna melanoma, sunlamp, sunbed

## Abstract

**Simple Summary:**

Motivated by the increasing incidences of skin cancer, in 2015, Australian states banned indoor tanning to prevent exposure to artificial ultraviolet light. However, there has been no study investigating the association between indoor tanning and early-onset melanoma and non-melanoma skin cancer. In this study, we reviewed a total of 54 studies to examine the association between indoor tanning device use and overall and early-onset skin cancer. We found that indoor tanning is associated with increased risk for early-onset melanoma and NMSC, and has a dose–response relationship with first exposure at an early age and the frequency of exposure. Therefore, this study emphasizes the importance of avoiding indoor tanning risk in younger adults. Our findings provide evidence that supports policies regulating the excessive use of tanning devices, especially in the vulnerable younger population, to reduce the additional risk of skin cancer.

**Abstract:**

The aim of this study was to examine the association between indoor tanning use and the risk of overall and early-onset (age < 50) melanoma and non-melanoma skin cancer (NMSC). To evaluate the association between indoor tanning and skin cancer, a systematic review of the literature published until July 2021 was performed using PubMed, EMBASE, and MEDLINE. Summary relative risk (RR) from 18 studies with 10,406 NMSC cases and 36 studies with 14,583 melanoma cases showed significant association between skin cancer and indoor tanning (melanoma, RR= 1.27, 95% CI 1.16–1.39; NMSC, RR = 1.40, 95% CI 1.18–1.65; squamous cell carcinoma (SCC), RR = 1.58, 95% CI 1.38–1.81; basal cell carcinoma (BCC), RR = 1.24, 95% CI 1.00–1.55). The risk was more pronounced in early-onset skin cancer (melanoma, RR = 1.75, 95% CI 1.14–2.69; NMSC, RR = 1.99, 95% CI 1.48–2.68; SCC, RR = 1.81, 95% CI 1.38–2.37; BCC, RR = 1.75, 95% CI 1.15–2.77). Moreover, first exposure at an early age (age ≤ 20 years) and higher exposure (annual frequency ≥ 10 times) to indoor tanning showed increasing risk for melanoma (RR = 1.47, 95% CI 1.16–1.85; RR = 1.52, 1.22–1.89) and NMSC (RR = 2.02, 95% CI 1.44–2.83; RR = 1.56, 95% CI 1.31–1.86). These findings provide evidence supporting primary prevention policies regulating modifiable behaviors to reduce the additional risk of skin cancer among younger adults.

## 1. Introduction

Skin cancer, including melanoma and non-melanoma skin cancer (NMSC), is the fifth most commonly occurring cancer, with over 1 million diagnoses worldwide and incidence rates continuing to increase rapidly [1,2]. The sharpest increase in risk is observed in people younger than 40 years of age [3,4,5]. These cases of early-onset skin cancer may represent gene–environment interaction, particularly among individuals with genetic susceptibility [6,7]. As a result, environmental factors may play an important role in primary prevention for skin cancer in younger adults. 

Exposure to ultraviolet (UV) radiation is a major environmental factor for melanoma and NMSC. According to previous studies, almost 90% of all melanomas, 85% of squamous cell carcinoma (SCC), and 82% of basal cell carcinoma (BCC) were attributable to excess UV radiation [3,8]. According to the recent monography from the International Agency for Research on Cancer (IARC), solar UV and the use of UV emitting tanning devices were classified as group 1 carcinogens, which were carcinogenic to humans based on the evidence from epidemiological research [9]. For this reason, Australian states are set to ban indoor tanning to prevent exposure to artificial UV in 2015 [10]. A few studies have investigated the association of indoor tanning with early-onset melanoma and NMSC, including SCC and BCC [11,12]. Previous studies have tended to use any type of indoor tanning device to investigate the impact of indoor tanning on melanoma and NMSC. Only one study of melanoma has suggested that there is a different impact on the melanoma risk according to the tanning device type [13]. Moreover, no study has yet integrated and characterized the clinicopathological parameters, such as the histopathological diagnosis and anatomic site. Additional updated prospective studies and epidemiological studies have been published since then, which have provided an opportunity to explore some aspects of the relationship between indoor tanning and skin cancer histopathological subtypes. 

This systematic review aims to assess and delineate the indoor tanning device type, as well as the clinicopathological characteristics of indoor tanning-associated overall and early-onset melanoma and NMSC. 

## 2. Materials and Methods

### 2.1. Literature Search and Study Selection

This study searched publications published up to July 2021 using the databases PubMed, EMBASE, and MEDLINE. The inclusion criteria for eligibility were as follows: (1) for the outcome of interest, the following keywords were considered: “skin cancer”, “skin neoplasm”, “melanoma”, “squamous cell carcinoma”, “SCC”, “basal cell carcinoma”, and “BCC”; (2) for exposure, following keywords were used: “sunbed”, “sunlamp”, “sunbathing”, “tanning”, “UV”, “artificial UV”, “artificial light”, “solaria”, and “solarium”; and (3) providing data for the calculation of the relative risk (RR) or odds ratio (OR) with 95% confidence intervals (CIs). All the titles and abstracts of the searched papers were reviewed to identify potentially eligible studies, and, furthermore, all the references from previous meta-analyses on skin cancer written in English were carried out. Cohort and case–control studies published as original articles were also selected in this study. For cohort studies from the same population, an updated dataset was included in this study. All included studies examined patients given the diagnosis of NMSC or melanoma who were ever exposed to an indoor tanning device. This study was conducted by following the Preferred Reporting Items for Systematic Reviews and Meta-Analyses (PRISMA) [14]. This study was registered in the International Prospective Register of Systematic Reviews (PROSPERO; registration number 292386).

### 2.2. Statistical Analysis

The pooled relative risk (RR) and 95% confidence intervals (95% CIs) of each association were calculated based on the risk with the whole confounding adjustment. When no estimates were suggested, the calculated crude estimates with 95% CIs obtained from the reported data were included in this study. For studies with estimates for both SCC and BCC, combined RRs of SCC risk and BCC risk were calculated as RRs of NMSC.

The meta-analysis used the random effects models using the maximum likelihood estimation to estimate the summary RR. Statistical heterogeneity evaluated the heterogeneity of the studies using Higgins and Thompson’s I^2^ statistics. Funnel plots and Egger and Begg tests were used to assess potential publication bias derived from the validity of the estimates. All analyses were done with R version 4.0.2. 

The following characteristics were analyzed from each study: (1) onset age (overall and early-onset (diagnosis before age 50)), (2) histology (squamous cell carcinoma and basal cell carcinoma for NMSC; superficial spreading melanoma (SSM), nodular melanoma (NM), lentigo maligna melanoma (LM), and others for melanoma); (3) anatomic site (trunk, head and neck, and limbs); (4) type of indoor tanning device (sunbed vs. sunlamp); (5) study design (cohort vs. case–control study); (6) publication year (<2000 vs. ≥2000); (7) first exposure at early age to indoor tanning (<20 years vs. ≥20 years); (9) annual frequency of indoor tanning (<10 times vs. ≥10 times). 

## 3. Results

### 3.1. Selection of Eligible Studies

According to the research strategy, a total of 484 records were identified. Of those articles, 399 were excluded by titles and abstracts, and 85 full-text articles were reviewed to assess their eligibility. Twelve studies with different exposures and outcomes, ten studies that were not original articles, five studies with duplicated populations, and four descriptive studies were removed from the 85 full-text articles. 

**Figure 1:**
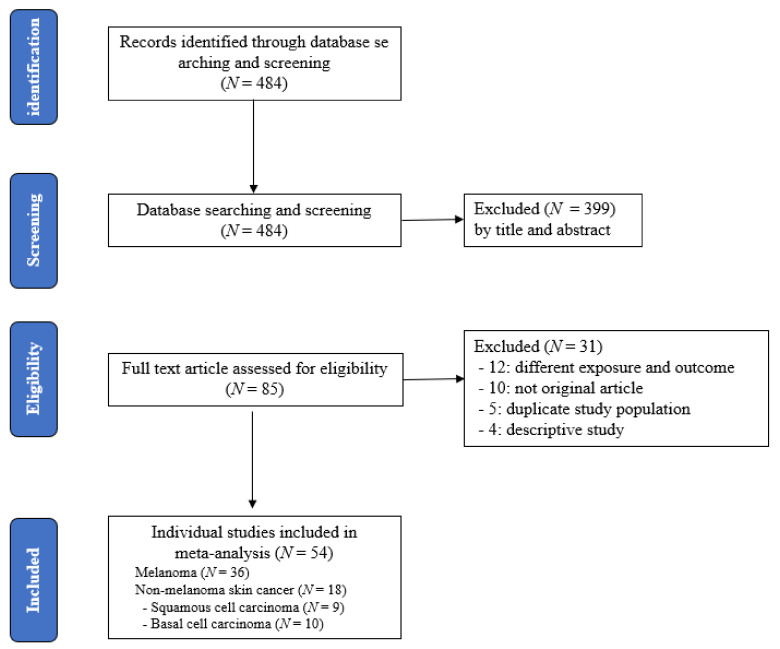
Flow chart according to the selection of studies related to risk of skin cancer by indoor tanning device use.

Finally, a total of 54 eligible articles (36 melanoma studies and 18 NMSC studies, including 9 SCC studies and 10 BCC studies) were selected for the meta-analysis. More details of publication selection for this meta-analysis are shown in Figure 1 (Tables S1 and S2). 

### 3.2. Main Analysis

**Figure 2:**
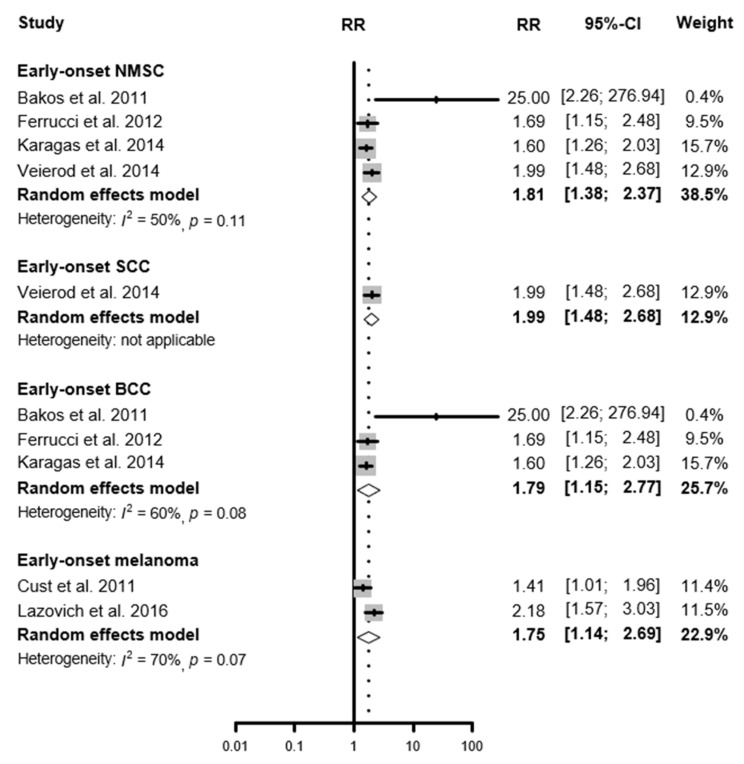
Relative risk for early-onset skin cancer associated with the ever-use of indoor tanning devices.

A total of 10,406 NMSC cases from 18 studies were included in this study. The summary RRs of indoor tanning device use were 1.40 (95% CI 1.18–1.65) for overall NMSC, 1.58 (95% CI 1.38–1.81) for SCC, and 1.24 (95% CI 1.00–1.55) for BCC. Based on the 36 studies including 14,583 melanoma cases, the summary RR of indoor tanning device use was 1.27 (95% CI 1.16–1.39) for overall cutaneous melanoma. These associations were even greater for early-onset skin cancer, defined as melanoma and NMSC occurring under the age of 50. The summary RR for early-onset NMSC was 1.81 (95% CI 1.38–2.37), for SCC was 1.99 (95% CI 1.48–2.68), and for BCC was 1.79, (95% CI 1.14–2.69). The summary RR for early-onset melanoma was 1.75 (95% CI 1.14–2.69) compared with never-users (Table 1, Figure 2, Figures S1 and S2). 

### 3.3. Sub-group Analysis

According to the type of indoor tanning device, both sunlamp (RR = 1.72, 95% CI 1.16–2.53) and sunbed use (RR = 1.48, 95% CI 1.20–1.83) were significantly associated with the increasing risk of SCC. For BCC, a significant association was observed in sunbed exposure (RR = 4.41, 95% CI 1.10–77.08), while no significant association was shown in sunlamp use (RR = 1.21, 95% CI 0.75–1.95) (Table 2 and Figures S3–S5). After stratifying the study design, a significant association was observed in the cohort studies for NMSC (RR = 1.48, 95% CI 1.18–1.84), while case–control studies had no significant association (RR = 1.27, 95% CI 0.97–1.72) (Table 2 and Figures S6 and S7). Among the NMSC studies, 3 were published before 2000 and 15 were published after 2000. We found a significant association for tanning device use with NMSC in the studies recruited after 2000 (RR = 1.39, 95% CI 1.17–1.66) (Table 2 and Figures S8 and S9). 

The anatomic sites of melanoma were classified by trunk, head and neck, and limbs. After stratifying the anatomic sites, ever-use of indoor tanning increased the risk of melanoma on the trunk (RR = 1.62, 95% CI 1.25–2.10) and on the limbs (RR = 1.38, 95% CI 1.07–1.77) (Table 2 and Figure S10). For histology classification of melanoma, there were two studies each on SSM and NM, and one each on LM and others. Among them, a significant association was observed between tanning devices and LM (RR = 2.83, 95% CI 1.37–5.84) (Table 2 and Figure S11). 

For melanoma, 28 of these 36 acceptable studies also provided a separate analysis of sunlamps and sunbeds. Both sunlamp (RR = 1.31, 95% CI 1.04–1.64) and sunbed exposure (RR = 1.17, 95% CI 1.05–1.31) were significantly associated with the risk of melanoma (Table 2). For an analysis restricted to the five cohort studies, the summary RR was 1.20 (95% CI 1.11–1.29) with no heterogeneity (I^2^ = 7%; *p* = 0.37) and 1.30 (95% CI 1.14–1.47) for 31 case–control studies. There was also a consistently significant association in before (RR = 1.28, 95% CI 1.09–1.50) and after 2000 (RR = 1.26, 95% CI 1.12–1.42) (Table 2 and Table S3). 

To assess the dose–response of indoor tanning, sub-group analyses were conducted, including first exposure at an early age to indoor tanning and the annual frequency of indoor tanning use. This study found that first exposure at an early age to indoor tanning was associated with 1.47-fold higher risk for melanoma (95% CI 1.16–1.85). High dose exposure to indoor tanning more than 10 times per year also related to an increasing risk of melanoma (RR = 1.52, 95% CI 1.22–1.89). For NMSC, the RR of first exposure at an early age was 2.02 (95% CI 1.44–2.83) for NMSC, 1.89 (95% CI 0.90–3.98) for SCC, and 1.86 (95% CI 1.44–2.41) for BCC. Compared to never-users, more than 10 tanning times a year had significantly increased the risk of NMSC (RR = 1.56, 95% CI 1.31–1.86), SCC (RR = 1.65, 95% CI 1.30–2.10), and BCC (RR = 1.46, 95% CI 1.28–1.66) (Table 3 and Figures S12 and S13). 

## 4. Discussion

This meta-analysis provides evidence of the association between indoor tanning and melanoma and non-melanoma skin cancer, particularly early-onset skin cancer diagnosed before age 50 years. These associations were stronger for melanoma occurring on the trunk and limbs. By stratifying the types of indoor tanning, this study found that both sunlamps and sunbeds were significantly associated with the risk of melanoma and squamous cell carcinoma. Moreover, first exposure at an early-age to indoor tanning, and frequent use of indoor tanning was significantly associated with an increased risk for melanoma and non-melanoma skin cancer. 

Although there are several previous meta-analyses of the association between indoor tanning device use and skin cancer, the association was mostly based on population-based studies [15,16,17,18,19]. 

In 2006, the IARC evaluated the dose–response relation between sunbed use and the risk of melanoma [15]. Although the study found that first exposure to sunbeds before the age of 35 years significantly increased the risk of melanoma, the IARC did not find significant evidence for the relation due to the limited number of studies. A recent meta-analysis, including large studies for melanoma published in 2018 [18] and for NMSC in 2012 [19], showed a significant association between indoor tanning and the risk of skin cancer. However, this association was mainly confirmed based on the case–control studies. Each meta-analysis included two cohort studies and only one cohort study, respectively. Although there are several meta-analyses of indoor tanning and melanoma and NMSC, there were limited studies conducted to document the association between indoor tanning and the risk of early-onset melanoma and NMSC. This meta-analysis, using more prospective studies [20,21,22,23,24,25,26,27], provided strong evidence of the causal relationship between indoor tanning and the risk of melanoma and NMSC, respectively. Notably, this association was stronger for the onset at an earlier age. A previous study reported that a young age group (<40 years), which was more frequently female, had more BRAF mutations and less NRAS mutations [28]. Although there are low cumulative UV doses in young adults, the higher prevalence of BRAF mutations is related to a higher risk of an earlier age of onset [29,30]. Based on the different mutational features between younger and older adults, different treatment is recommended to those of an earlier age of onset. 

The type of indoor tanning device is divided into sunbed and sunlamp. It is controversial that sunlamps and sunbeds have different effects on skin cancer. A population-based study showed modest association between sunlamp use and melanoma risk, whereas no association was found for sunbeds [31]. On the other hand, another multicenter study showed no significant difference in the effect on melanoma risk according to the tanning device type [32]. For NMSC, no epidemiological study has been conducted to compare sunbeds and sunlamps. In general, UVA (315–400 nm) is the primary source of sunbeds, while sunlamp use in homes emitted UVB (280–315 nm) [13]. UVB directly damages DNA, whereas UVA indirectly damages DNA because it has a longer wavelength which can penetrate deeper into the skin area and indirectly affect DNA-damaging [33]. Thus, both UVA and UVB damage cells and DNA, leading to initiate carcinogenesis of melanoma and NMSC. These biological backgrounds support our findings for the association between both sunbeds and sunlamps and melanoma and NMSC, especially SCC. For BCC, although only two papers about sunbeds and sunlamps were included in this study, respectively, the results showed that only sunbeds had a significant association with BCC. This finding suggests that the long wavelengths of UVA from the sunbed might cause BCC. Further research is needed to assess the potential effect of sunlamps and sunbeds on the risk of BCC. 

Melanoma and NMSC mainly have different anatomical locations where cancer occurs. Melanoma is commonly diagnosed in the trunk [34,35,36], while NMSC (SCC and BCC) is commonly on the head and/or neck [37,38]. This study showed consistent findings with previous studies. The different anatomical locations between melanoma and NMSC might be a variation in the parts of the body exposed to the UV and pathological differences in the tanning device type [29]. However, based on the limited studies of anatomical locations according to tanning device type, it was not possible to perform the meta-analysis of melanoma and NMSC by anatomical site and tanning type.

Besides, this meta-analysis confirmed the consistency that individuals who had first exposure at an early age to indoor tanning (<20 years) and had more than 10 tanning times in a year were associated with skin cancer risk. This is in line with the previous research that showed a dose–response relation between indoor tanning and the risk of melanoma and NMSC [21,22,39,40,41,42,43,44].

Our study has several limitations. First, individual studies included in this meta-analysis were concentrated in the population from Europe and North America. Second, due to the limited information of histological subtypes of melanoma in prior studies, the risk of histological melanoma subtypes from indoor tanning was observed as mostly insignificant in our meta-analysis. Third, the insufficient number of SCC and BCC studies with information on the tanning type. Although we found a significant association between sunbed usage and SCC and BCC, there may not be enough statistical power to verify the link. Further meta-analysis should be performed based on more studies in the future to verify this association for SCC and BCC. 

Nevertheless, this meta-analysis has several strengths. Unlike previous studies, this study presents the risk of early-onset and histological and anatomical subtypes by exposure to indoor tanning according to device type. 

## 5. Conclusions

In conclusion, this meta-analysis suggests indoor tanning increases the risk of skin cancer, particularly early-onset melanoma and NMSC. There are constantly increasing trends for skin cancer worldwide [2], especially in women at younger ages due to the social and economic factors related to tanning behavior [1,3,4,45]. The findings provide supporting evidence for prevention policies regulating modifiable behaviors to reduce the additional risk of skin cancer. Therefore, some guidelines or regulations on the use of tanning devices in younger adults should be actively formulated.

## Figures and Tables

**Table 1:** Meta-analysis for association with indoor tanning on the risk of skin cancer (overall and early-onset).

Skin Cancer	Study N	Summary RR (95% CI)	Heterogeneity	
			I^2^ (%)	P-Cochran
**Overall**				
Non-melanoma skin cancer ^1^	18	1.40 (1.18–1.65)	77	<0.01
Squamous cell carcinoma	9	1.58 (1.38–1.81)	27	0.21
Basal cell carcinoma	10	1.24 (1.00–1.55)	61	<0.01
Cutaneous melanoma	36	1.27 (1.16–1.39)	62	<0.01
**Early-onset (Age < 50 years) ^2^**				
Non-melanoma skin cancer	4	1.81 (1.38–2.37)	50	0.11
Squamous cell carcinoma	1	1.99 (1.48–2.68)	-	-
Basal cell carcinoma	3	1.79 (1.15–2.77)	60	0.08
Cutaneous melanoma	2	1.75 (1.14–2.69)	70	0.07

^1^ Non-melanoma skin cancer studies including two studies for NMSC, six for SCC, seven for BCC, and three for both SCC and BCC. ^2^ Diagnosed with skin cancer at age < 50 years.

**Table 2:** Summary of relative risks by sub-group analyses on indoor tanning and melanoma and non-melanoma skin cancer (NMSC).

Ever Use of Indoor Tanning Device	Study N	Summary RR (95% CI)	Heterogeneity	
			I^2^ (%)	P-Cochran
**Non-melanoma skin cancer**				
Histology				
Squamous cell carcinoma	9	1.58 (1.38–1.81)	27	0.21
Basal cell carcinoma	10	1.24 (1.00–1.55)	61	<0.01
Anatomic site				
Not defined in each paper	18	1.40 (1.18–1.65)	77	<0.01
Type of indoor tanning				
NMSC, overall ^1^				
Sunlamp	4	1.22 (0.75–1.97)	58	0.07
Sunbed	7	1.43 (1.10–1.85)	82	<0.01
SCC				
Sunlamp	2	1.72 (1.16–2.53)	0	0.47
Sunbed	5	1.48 (1.20–1.83)	61	0.04
BCC				
Sunlamp	2	1.21 (0.75–1.95)	0	0.95
Sunbed	2	4.41 (1.10–77.08)	83	0.02
Study design				
Cohort ^2^	5	1.48 (1.18–1.84)	83	<0.01
Case-control study (NMSC, overall)	13	1.29 (0.97–1.72)	70	<0.01
(SCC)	5	1.76 (1.22–2.52)	51	0.09
(BCC)	9	1.17 (0.84–1.64)	65	<0.01
Publication year				
<2000 ^3^	3	1.57 (0.75–3.27)	50	0.13
≥2000 (NMSC, overall)	15	1.39 (1.17–1.66)	79	<0.01
(SCC)	7	1.55 (1.25–1.92)	73	<0.01
(BCC)	8	1.19 (0.90–1.56)	74	<0.01
**Cutaneous melanoma**				
Histology				
Superficial spreading melanoma (SSM)	2	1.21 (0.81–1.82)	75	0.05
Nodular melanoma (NM)	2	1.03 (0.51–2.11)	66	0.09
Lentigo maligna melanoma (LM)	1	2.83 (1.37–5.84)	-	-
Others	1	1.27 (0.63–2.58)	-	-
Anatomic site				
Trunk	6	1.62 (1.25–2.10)	57	0.04
Head and neck	5	1.16 (0.64–2.13)	68	0.01
Limbs	5	1.38 (1.07–1.77)	55	0.07
Type of indoor tanning				
Sunlamp	9	1.31 (1.04–1.64)	52	0.03
Sunbed	19	1.17 (1.05–1.31)	56	<0.01
Study design				
Cohort	5	1.20 (1.11–1.29)	7	0.37
Case-control study	31	1.30 (1.14–1.47)	65	<0.01
Publication year				
<2000	15	1.28 (1.09–1.50)	35	0.09
≥2000	21	1.26 (1.12–1.42)	72	<0.01

Abbreviation: P-Cochran, *p*-value in Cochran Q test; SCC, Squamous cell carcinoma; BCC, Basal cell carcinoma; NMSC, Non-melanoma skin cancer. ^1^ Three studies had the results of both SCC and BCC risks (Zhang et al. (2012); Bajdik et al. (1996); Han et al. (2006)). We calculated summary RRs for SCC risk and BCC risk, and the calculated summary RRs were included in the meta-analysis. ^2^ Four of five cohort studies were focused on SCC risk. ^3^ Two of three cohort studies were focused on SCC risk and two of three studies were focused on BCC risk.

**Table 3:** First exposure at early age to indoor tanning, annual frequency of indoor tanning use, and the risk of melanoma and non-melanoma skin cancer (NMSC).

Exposure	Study N	Summary RR (95% CI)	Heterogeneity	
			I^2^ (%)	*p*-Value
**Cutaneous melanoma**				
First exposure at early age to indoor tanning (year) ^1^				
<20	9	1.47 (1.16–1.85)	61	<0.01
≥20	9	1.28 (1.01–1.63)	77	<0.01
Annual frequency of indoor tanning (times) ^2^				
<10	6	1.33 (1.00–1.78)	60	0.03
≥10	6	1.52 (1.22–1.89)	0	0.67
**Non-melanoma skin cancer**				
First exposure at early age to indoor tanning (year) ^3^				
<20	6	2.02 (1.44–2.83)	68	<0.01
≥20	6	1.48 (1.31–1.68)	0	0.56
Annual frequency of indoor tanning (times) ^4^				
<10	3	1.32 (1.14–1.52)	40	0.19
≥10	3	1.56 (1.31–1.86)	41	0.18
**Squamous cell carcinoma**				
First exposure at early age to indoor tanning (year)				
<20	3	1.89 (0.90–3.98)	75	0.02
≥20	3	1.53 (1.26–1.85)	27	0.25
Annual frequency of indoor tanning (times)				
<10	2	1.46 (1.24–1.71)	0	0.97
≥10	2	1.65 (1.30–2.10)	10	0.29
**Basal cell carcinoma**				
First exposure at early age to indoor tanning (year)				
<20	2	1.86 (1.44–2.41)	0	0.61
≥20	2	1.51 (1.19–1.92)	0	0.69
Annual frequency of indoor tanning (times)				
<10	2	1.29 (1.01–1.65)	40	0.20
≥10	2	1.46 (1.28–1.66)	18	0.27

^1^ For Swerdlow et al. in 1988, an estimate of age at first indoor tanning device use <30 years and ≥30 years; for Westerdahl et al. in 2000, an estimate of age at first indoor tanning device use ≤35 years and >35 years; for Lazovich et al. in 2010, an estimate of age at first indoor tanning device use <18 years and ≥18 years; for Chen et al. in 1998, Cust et al. in 2011, and Elliott et al. in 2012, an estimate of age at first indoor tanning device use <25 years and ≥25 years; for Farley et al. in 2015, an estimate of age at first indoor tanning device use ≤19 years and >19 years were used. ^2^ For Zhang et al. in 2012, an estimate of the number of tanning device use <6 times/year and 6+ times/year; for Stenehjem et al. in 2017, an estimate of the number of indoor tanning device use 1–2 times/month and 3–5 times/month; for Ghiasvand et al. in 2019, an estimate of the number of indoor tanning device use <15 times/lifetime and 15+ times/lifetime were used. ^3^ For Ferrucci et al. in 2012, an estimate age at first indoor tanning device use ≤18 years and >18 years; and for Simon Lergenmuller et al. in 2019 an estimate age at first indoor tanning device use <30 years and ≥18 years were used. ^4^ For Ferrucci et al. in 2012, an estimate of 1–18 indoor tanning sessions and >18 sessions; and for Zhang et al. in 2012, an estimate of ≤6 times/year and >6 times/year were used.

## Data Availability

The data presented in this study are openly available at https://www.mdpi.com/journal/cancers.

## Supplementary Materials

The following are available online at https://www.mdpi.com/article/10.3390/cancers13235940/s1, Figure S1: Meta-analysis for risk of melanoma due to indoor tanning device use, Figure S2: Meta-analysis for risk of non-melanoma skin cancer (NMSC) due to indoor tanning device use, Figure S3: Meta-analysis for risk of non-melanoma skin cancer (NMSC) due to different indoor tanning device (sunlamps vs. sunbeds), Figure S4: Meta-analysis for risk of squamous and basal cell skin cancer (SCC and BCC) due to different indoor tanning device (sunlamps vs. sunbeds), Figure S5: Meta-analysis for risk of melanoma due to different indoor tanning device (sunlamps vs. sunbeds), Figure S6: Meta-analysis according to study design for risk of melanoma due to indoor tanning device use, Figure S7: Meta-analysis according to study design for risk of non-melanoma skin cancer (NMSC) due to indoor tanning device use, Figure S8: Meta-analysis according to publication period for risk of melanoma due to indoor tanning device use, Figure S9: Meta-analysis according to publication period for risk of non-melanoma skin cancer (NMSC) due to indoor tanning device use, Figure S10: Meta-analysis for risk of melanoma anatomic subtypes due to indoor tanning device use, Figure S11: Meta-analysis for risk of melanoma histological subtypes due to indoor tanning device use, Figure S12: Meta-analysis for risk of melanoma due to age at first indoor tanning device use, Figure S13: Meta-analysis for risk of melanoma due to the number of indoor tanning device use per year, Table S1: Studies for association between non-melanoma skin cancer (NMCS) and indoor tanning included in meta-analysis, Table S2: Studies for association between melanoma and indoor tanning included in meta-analysis, Table S3: Number of individuals diagnosed with skin cancer by subgroup.
