# Supplementary material for: Indoor Tanning and the Risk of Overall and Early-Onset Melanoma and Non-Melanoma Skin Cancer: Systematic Review and Meta-Analysis
Source: Cancers (Basel). 2021 Nov 25;13(23):5940. doi: 10.3390/cancers13235940 (PMC8656707; doi:10.3390/cancers13235940)
Supplement: Supplementary file 1 [file cancers-13-05940-s001.zip › cancers-1456849-supplementary.pdf]

Table S1. Studies for association between non-melanoma skin cancer (NMCS) and indoor tanning included in meta-analysis

1st Author(year)	Country	Study period	Study population	Female (%)	Age (years)	Cohort (person-year) or Control	Cases			Adjustment
**Cohort study**							**SCC**	**BCC**	**NMSC N/D**	
Zhang et al. (2012) (20)	USA	1989-2009	Nurses' Health Study II (NHSII)	100	25-42	73,145 (1,360,402) 97,134 (1,905,614)	403	5,506		Age, family history of melanoma, hair color at age 20, No. of moles on the legs, childhood tendency to sunburn, No. of sunburns, outdoor exposure, and UV index in the region of residence
Veierod et al. (2014) (21)	Norway, Sweden	1991-2009	Norwegian-Swedish Women's Lifestyle and Health Cohort study	100	30-50	1,584 (N/A)	131			Age, region of residence, and hair color and skin color after sun exposure
Stenehjem et al. (2017) (22)	Sweden	1998-2012	Cancer Registry of Norway	0	20-84	29,088 (N/A)			109	Age, benzene exposure duration, and education
Christensen et al. (2019) (23)	Sweden	1990-2011	Melanoma Inquiry of Southern Sweden (MISS)	100	25-85	113,290 (2,279,075)	328			Crude
Simon Lergenmuller et al. (2019) (45)	Norway, Sweden	1991-2015	Norwegian Women and Cancer Study	100	N/A	113,290	366			Age, UV index in the region of residence, hair color, and cumulative numbers of sunburns and sunbathing vacation
**Case-control study**										
Aubry et al. (1985) (46)	Canada	1977-1978	A hospital in the city of Montreal or Westmount	34.1	N/A	174	92			N/A
Bajdik et al. (1996) (47)	Canada	1983-1984	Alberta Cancer Registry	N/A	N/A	404	226	180		Age, ethnic origin, skin and hair color, and occupational sun exposure
Rosso et al. (1999) (48)	Switzerland	1994-1996	Switzerland	47.6	20-75	144		120		Age and sex
Corona et al. (2001) (49)	Italy	1995-1997	Hospitals for skin diseases in Rome, Italy	52.2	18-N/A	158		166		Age, sex, pigmentary traits, family history of skin cancer, outdoor work, and number of weeks spent at the beach before the age of 20 years

Karagas et al. (2002) (50)	USA	1993-1995	New Hampshire Skin Cancer Study	N/A	25-74	539	292			Age, sex, and sensitivity
Han et al. (2006) (51)	USA	1998-2000	Nurses' Health Study (NHS)	100	30-55	804	275	283		Constitutional susceptibility score, family history of skin cancer, sunburns, and sun exposure
Asgari et al. (2011) (52)	USA	2004-2005	Kaiser Permanente Northern California	38.1	43-85	830	415			Crude
Bakos et al. (2011) (53)	Germany	2004-2008	N/A	N/A	19-40	25		25		sun habits, medical conditions, and lifestyle-related variables
Gon and Minelli et al. (2011) (54)	Brazil	2006-2007	Dermatology center in southern Brazil	583	18-80	407		127		Age, sex, eye color, hair color, skin type, family history of skin cancer, and presence of actinic keratosis
Ferrucci et al. (2012) (42)	USA	2007-2010	Yale Study of Skin Health	69.2	N/A-40	370		367		Age, sex, body site, skin color, family history of melanoma, tanning ability, and MC1R non-synonymous variants
Karagas et al. (2014) (43)	USA	1993-1995, 1997-2000	New Hampshire Skin Cancer Study	60.8	25-50	452		657		Age, sex, tanning ability, and study phase
Kaskel et al. (2015) (55)	Germany	1997-1999	Germany	50.0	N/A	329		212		Crude
Larese Filon et al. (2019) (56)	Italy	2015-2016	University of Trieste	62.6	25-75	187			126	Crude

Abbreviations: N/A, Not available; SCC, Squamous cell carcinoma; BCC, Basal cell carcinoma; NMSC N/D, Non-melanoma skin cancer without histological classification

Table S2. Studies for association between melanoma and indoor tanning included in meta-analysis

1st Author(year)	Country	Study period	Study population	Female (%)	Age (years)	Cohort (person-year) or Control	Cases[1]	Adjustment
**Cohort study**								
Veierod et al. (2010) (25)	Norway, Sweden	1991-2005	Norwegian-Swedish Women's Lifestyle and Health Cohort study	100	30-50	105,954 (1,489,298)	412	Age, region of residence, pigmentation characteristics (hair color and skin color after heavy sun exposure), and solar exposure
Nielsen et al. (2012) (26)	Sweden	1990-2007	Melanoma Inquiry of Southern Sweden (MISS)	100	25-64	29,310 (458,950)	210	Heredity, nevi, hair color, freckles, sunburns, blisters, ulcers, sun vacations winter, and sunbathing vacation
Zhang et al. (2012) (20)	USA	1989-2009	Nurses' Health Study II (NHSII)	100	25-42	73,145 (1,360,402)	349	Age, family history of melanoma, hair color at age 20, No. of moles on the legs, childhood tendency to sunburn, No. of sunburns, outdoor exposure, and UV index in the region of residence
Stenehjem et al. (2017) (22)	Sweden	1998-2012	Cancer Registry of Norway	0	20-84	1,584 (N/A)	109	Age, benzene exposure duration, and education
Ghiasvand et al. (2019) (27)	Norway, Sweden	1991-2015	Norwegian-Swedish Women's Lifestyle and Health Cohort study	100	30-75	161,540 (2,682,000)	1,310	Age, birth cohort, skin color, hair color, education, and UV index in the region of residence
**Population-based case control study**								
Adam et al. (1981) (57)	UK	1971-1976	Oxford and South Western cancer registries	100	15-49	342	111	N/A
Holman et al. (1986) (58)	Australia	1980-1981	Western Australia	N/A	N/A	511	511	N/A
Elwood et al. (1986) (59)	UK	1981-1984	Nottinghamshire healthcare Service (NHS)	70.0	18-82	87	83	N/A
Osterlind et al. (1988) (60)	Denmark	1982-1985	East Denmark	N/A	20-79	926	474	Sex, naevi, freckles, and hair color
Zanetti et al. (1988) (61)	Italy	1984-1986	Province of Torino	N/A	N/A	416	208	Age, education, hair color, sunburns, and sun sensitivity

Swerdlow et al. (1988) (62)	UK	1979-1984	University departments of dermatology in Glasgow and Edinburgh and the west of Scotland plastic surgery	N/A	15-84	197	180	Crude
Mackie et al. (1989) (63)	UK	1987	Scottish Melanoma Group and Scottish Cancer Registry records	64.6	11-N/A	280	280	Sex, freckles, naevi, region of residence, and skin color
Garbe et al. (1993) (64)	Europe	1983-1990	German Dermatological Society	N/A	N/A	705	856	Nevi, hair type, and sensitivity to sunlight
Dunn-Lane et al. (1993) (65)	Ireland	1985-1986	Dublin hospitals	29	15-82	100	100	Crude
Westerdahl et al. (1994) (66)	Sweden	1988-1990	South Swedish Health Care Region	53.4	15-75	640	400	Sunburns, hair color, nevi, and history of sunbathing
Autier et al. (1994) (67)	Europe	1991-N/A	Belgium: Oeuvre Belge du Cancer; France: Conseil General du Rhone; Germany: Hamburg	55.0	20-N/A	447	420	Age, sex, ate at leaving school, area, and hair color
Holly et al. (1995) (68)	USA	1981-1986	Five San Francisco Bay Area countries	100	25-59	930	452	Crude
Chen et al. (1998) (13)	USA	1987-1989	Cancer Prevention Research Unit (CPRU)	N/A	N/A	512	624	Age, sex cutaneous phenotype index (hair color, eye color, and skin type or tanning ability), and sun exposure
Wolf et al. (1998) (69)	Austria	N/A	Styria, Austria			316	192	Age and sex
Walter et al. (1999) (70)	Canada	1984-1986	Resident of one of six countries in Sothern Ontario	52.5	20-69	608	583	Age, sex and tanning ability
Westerdahl et al. (2000) (71)	Sweden	1995-1997	South Swedish Health Case Region	50.2	16-80	913	571	Hair color, nevi, skin type and sunburns
Naldi et al. (2000) (72)	Italy	1992-1995	Italian Group for Epidemiology Research in Dermatology (GISED)	57.8	35-65	538	542	Age, sex, education, eye color, freckles, hair color, marital status, naevi, skin color, sunburns, vacations
Vajdix et al. (2004) (73)	Australia	1996-1998	Cancer registries	37.2	18-79	893	246	Age, sex, place of birth, eye color, tanning ability, and sun exposure
Bataille et al. (2004) (74)	UK	1989-1993	North East Thames region of UK	N/A	16-75	416	413	Age, sex, skin type, cumulative lifetime numbers of weeks abroad in hot countries, and total numbers of sunburns
Bataille et al. (2005) (32)	Europe	1988-2001	Sweden: Karolinska Institute in Stockholm and Uppsala hospital; Netherlands: Daniel den Hoed Cancer Center in Rotterdam and general	64.5	18-49	622	597	Age, sex, and skin phenotype

			hospitals from South of the Netherlands;<br>UK: Hospitals from Greater London area;<br>Belgium: Jules Bordet Institute, Brussels;<br>France: Cancer hospitals throughout France (Burgundy, Lille, Thionville, Paris and Bordeaux)					
Han et al. (2006) (51)	USA	1998-2000	Nurses' Health Study (NHS)	100	30-55	804	200	Constitutional susceptibility score, family history of skin cancer, sunburns, and sun exposure
Ting et al. (2007) (75)	USA	N/A	Academic dermatology clinic	61.2	N/A	1,439	79	Age, sex, race, tan ability, education, work environment, history of sunburn, and number of sunburns
Clough-Gorr et al. (2008) (31)	USA	1995-1998	Dartmouth College	47.3	20-69	678	423	Age, sex, family history of melanoma, hair color, freckles, sun sensitivity, and sun exposure
Lazovich et al. (2010) (41)	USA	2004-2007	Skin Health Study	59.9	25-59	1,101	1,167	Age, sex, eye color, hair color, skin color, freckle, moles, income, education, family history of melanoma, sun exposure, outdoor and occupational sun exposure, sunscreen use, and sunburns
Cust et al. (2011) (40)	Australia	2000-2002	Sydney, Melbourne and Brisbane, which are urban population in Australia	60.1	18-39	479	604	Age, sex, region of recruitment, education, family history of melanoma, tanning ability, and sun exposure
Fears et al. (2011) (39)	USA	1991-1992	United States	47.7	20-79	945	718	N/A
Zivkovic et al. (2012) (76)	Croatia	2010-2011	University Hospital 'Sestre milosrdnice'	45.8	18-65	120	120	Crude
Elliott et al. (2012) (77)	UK	2000-2005	Leeds Melanoma Study	N/A	17-76	855	483	Age, sex, educational level, family history of melanoma, sun sensitivity and cumulative lifetime total sun exposure
Kaskel et al. (2015) (55)	Germany	1997-1999	Germany	50.0	N/A	329	291	Crude
Farley et al. (2015) (38)	USA	2001-2013	Emory University	56.5	18-50	195	265	Sex, family history of melanoma, hair color, eye color, and tanning ability
Lazovich et al. (2016) (78)	USA	2004-2007	Skin Health Study	68.3	25-49	654	681	Crude

Abbreviations: N/A, Not Available

Table S3. Number of individuals diagnosed with skin cancer by subgroup

	Cases, N
**Non-melanoma skin cancer**	10,406
Histology	
Squamous cell carcinoma	2,528
Basal cell carcinoma	7,643
Type of indoor tanning	
NMSC, overall	
Sunlamp	944
Sunbed	8,100
SCC	
Sunlamp	678
Sunbed	1,927
BCC	
Sunlamp	145
Sunbed	5,531
Study design	
Cohort	6,843
Case-control study	3,563
Publication year	
< 2000	618
≥ 2000 (NMSC, overall)	9,788
**Cutaneous melanoma**	14,583
Histology	
Superficial spreading melanoma (SSM)	769
Nodular melanoma (NM)	132
Lentigo maligna melanoma (LM)	41
Others	50
Anatomic site	
Trunk	1,488
Head and neck	304
Limbs	1,738
Type of indoor tanning	
Sunlamp	5,337
Sunbed	8,215
Study design	
Cohort	2,390
Case-control study	12,193
Publication year	
< 2000	5,474

≥ 2000	9,109

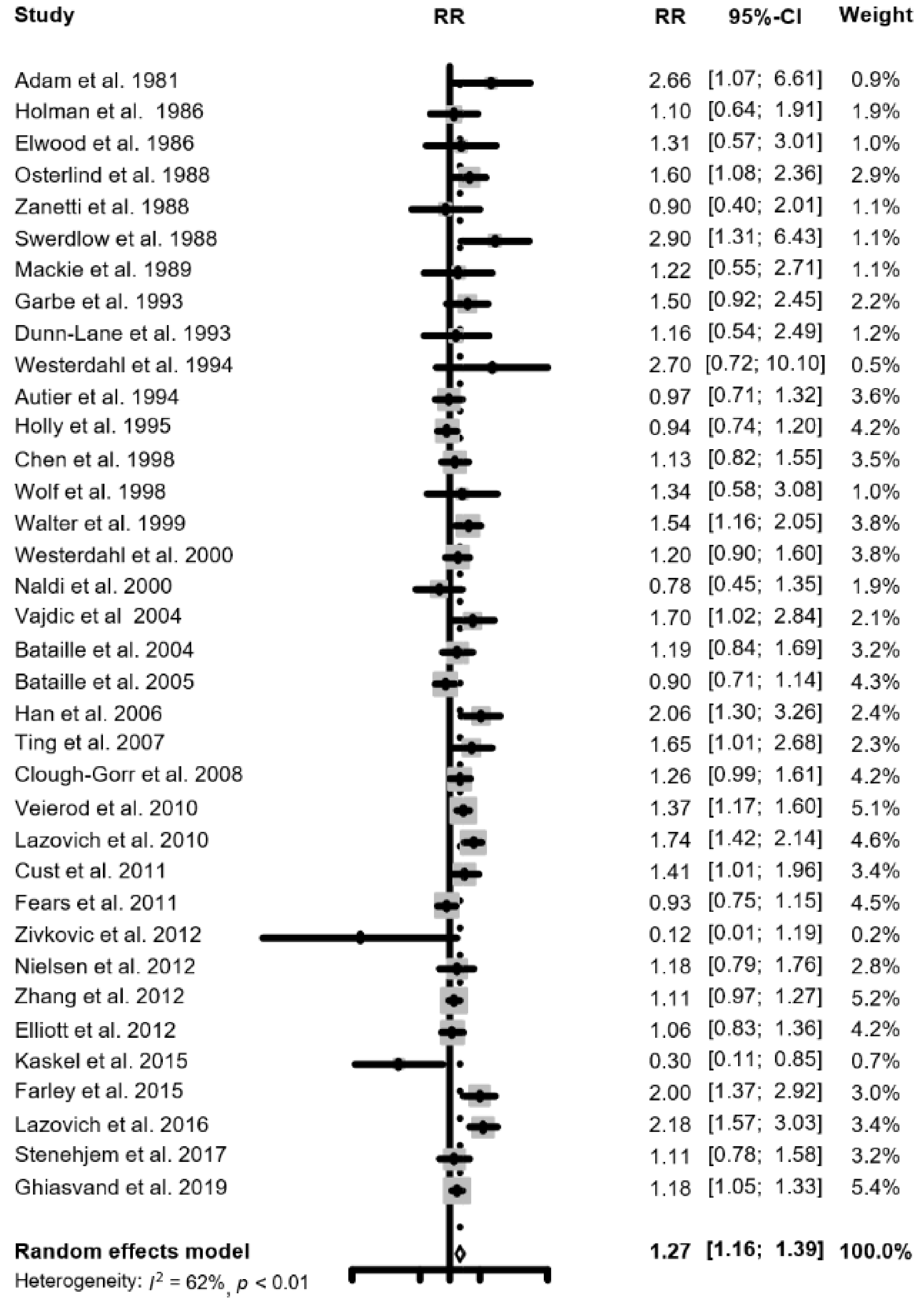

Figure S1. Meta-analysis for risk of melanoma due to indoor tanning device use.

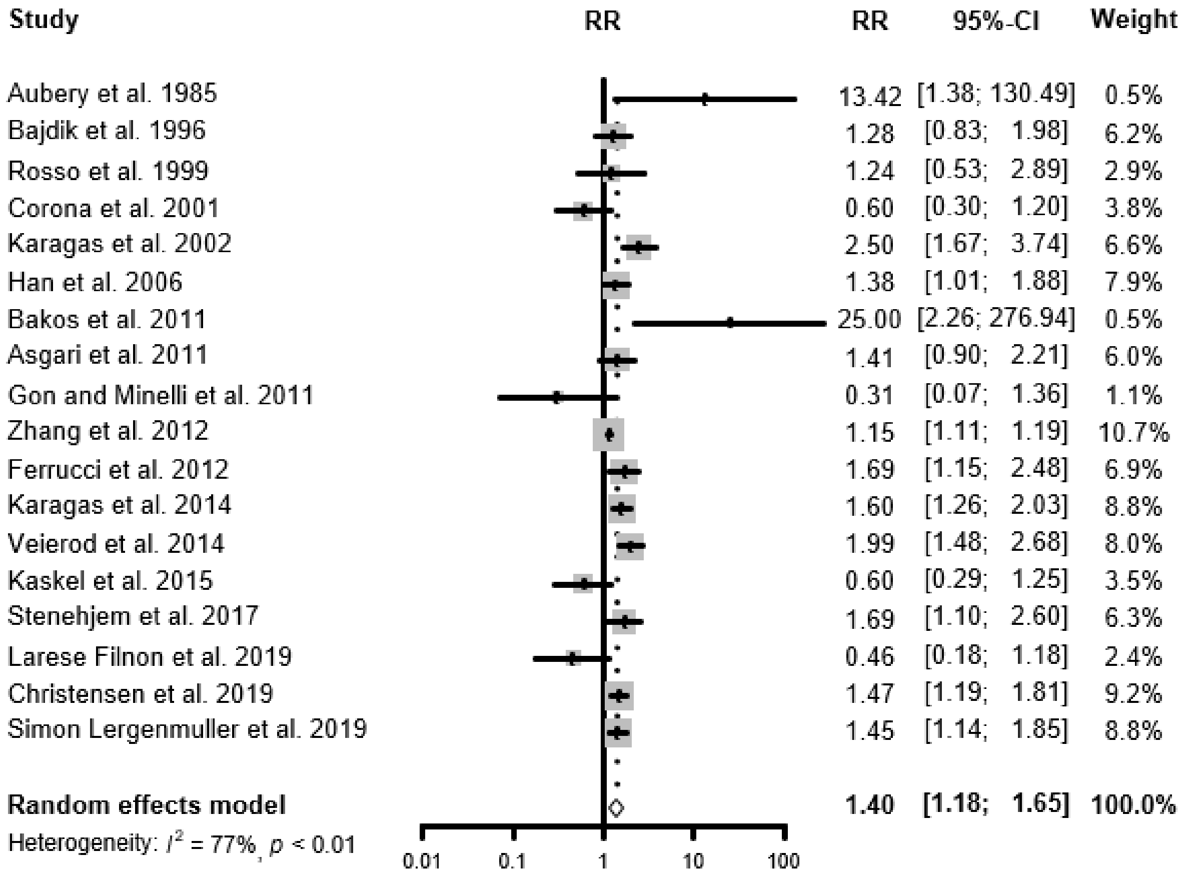

Figure S2. Meta-analysis for risk of non-melanoma skin cancer (NMSC) due to indoor tanning device use.

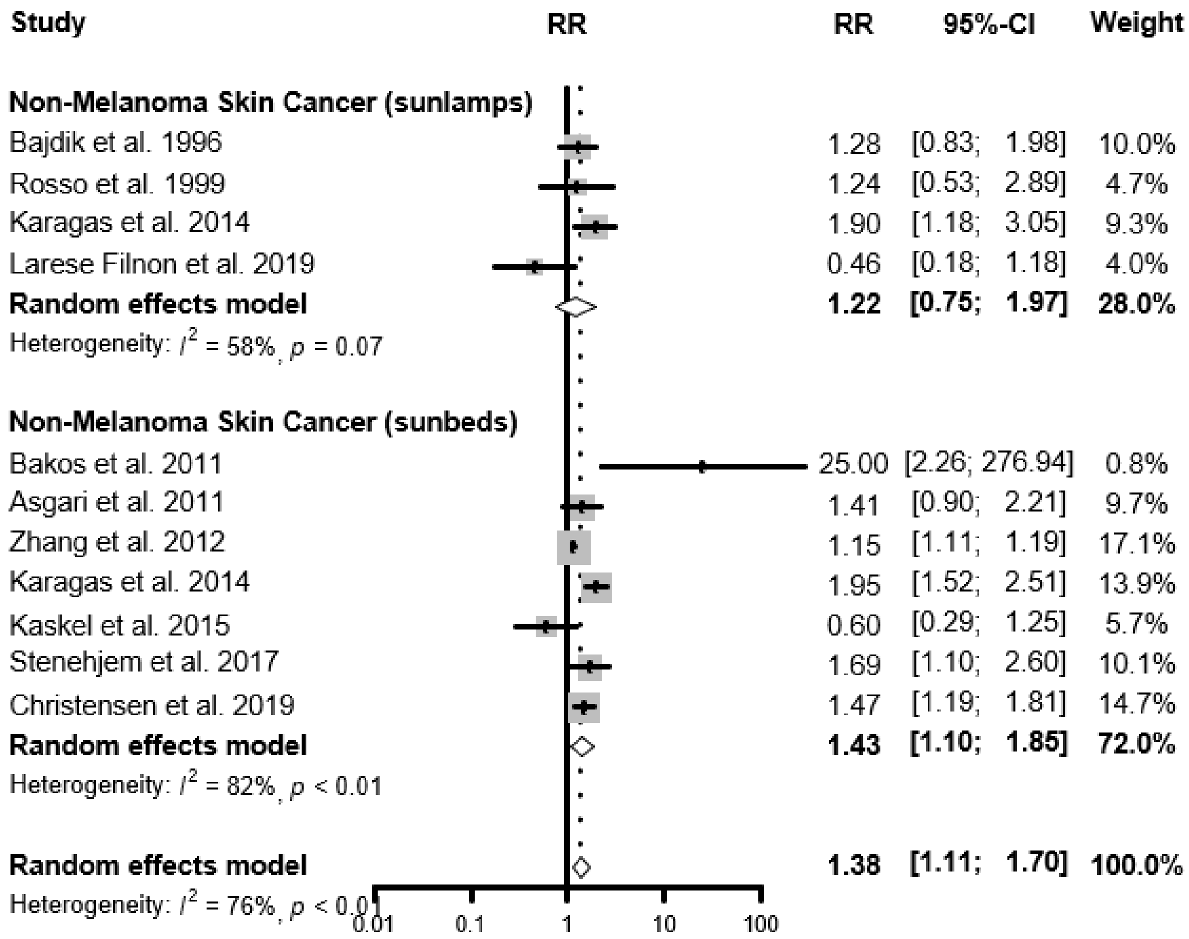

Figure S3. Meta-analysis for risk of non-melanoma skin cancer (NMSC) due to different indoor tanning device (sunlamps vs. sunbeds).

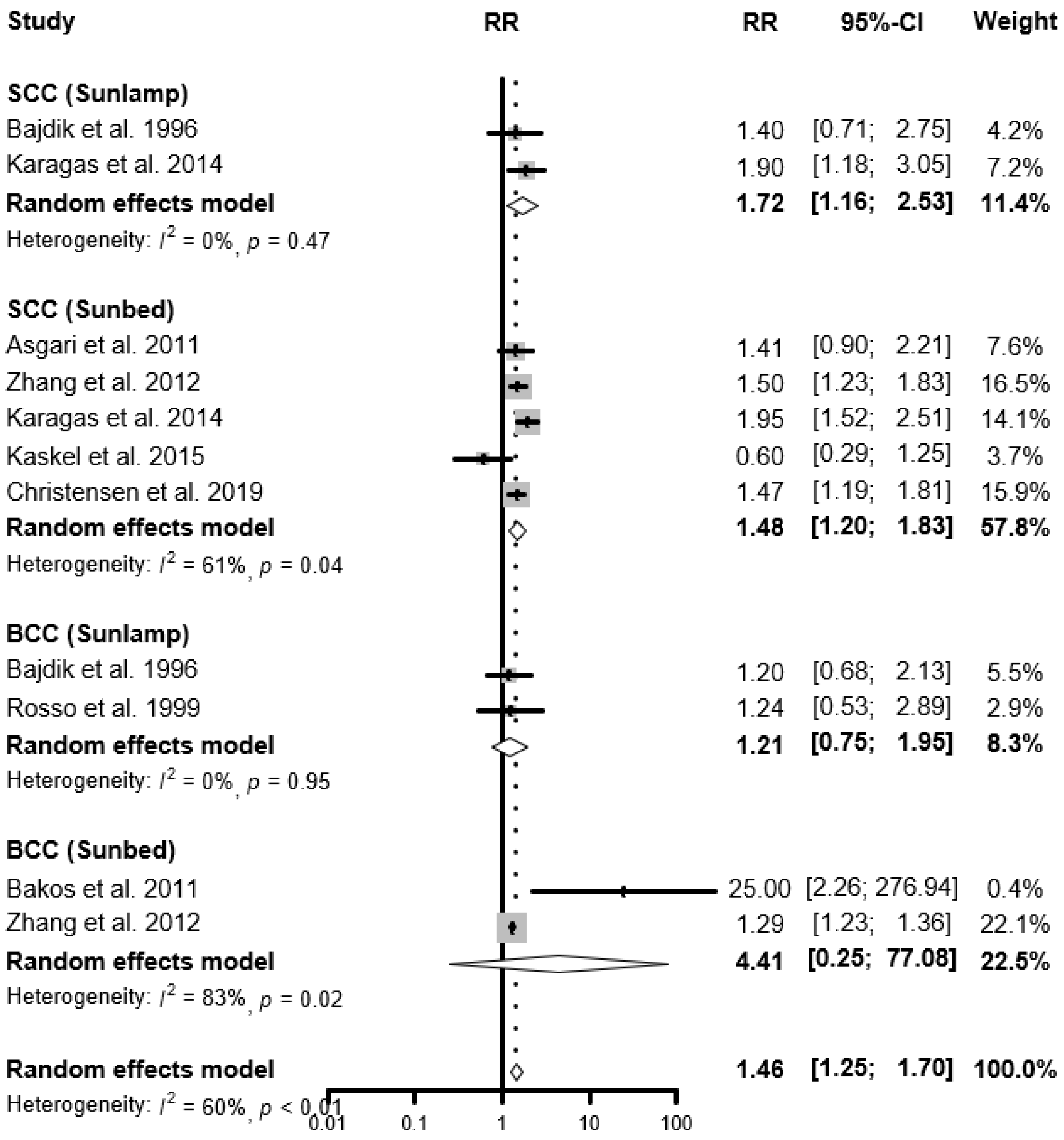

Figure S4. Meta-analysis for risk of squamous and basal cell skin cancer (SCC and BCC) due to different indoor tanning device (sunlamps vs. sunbeds).

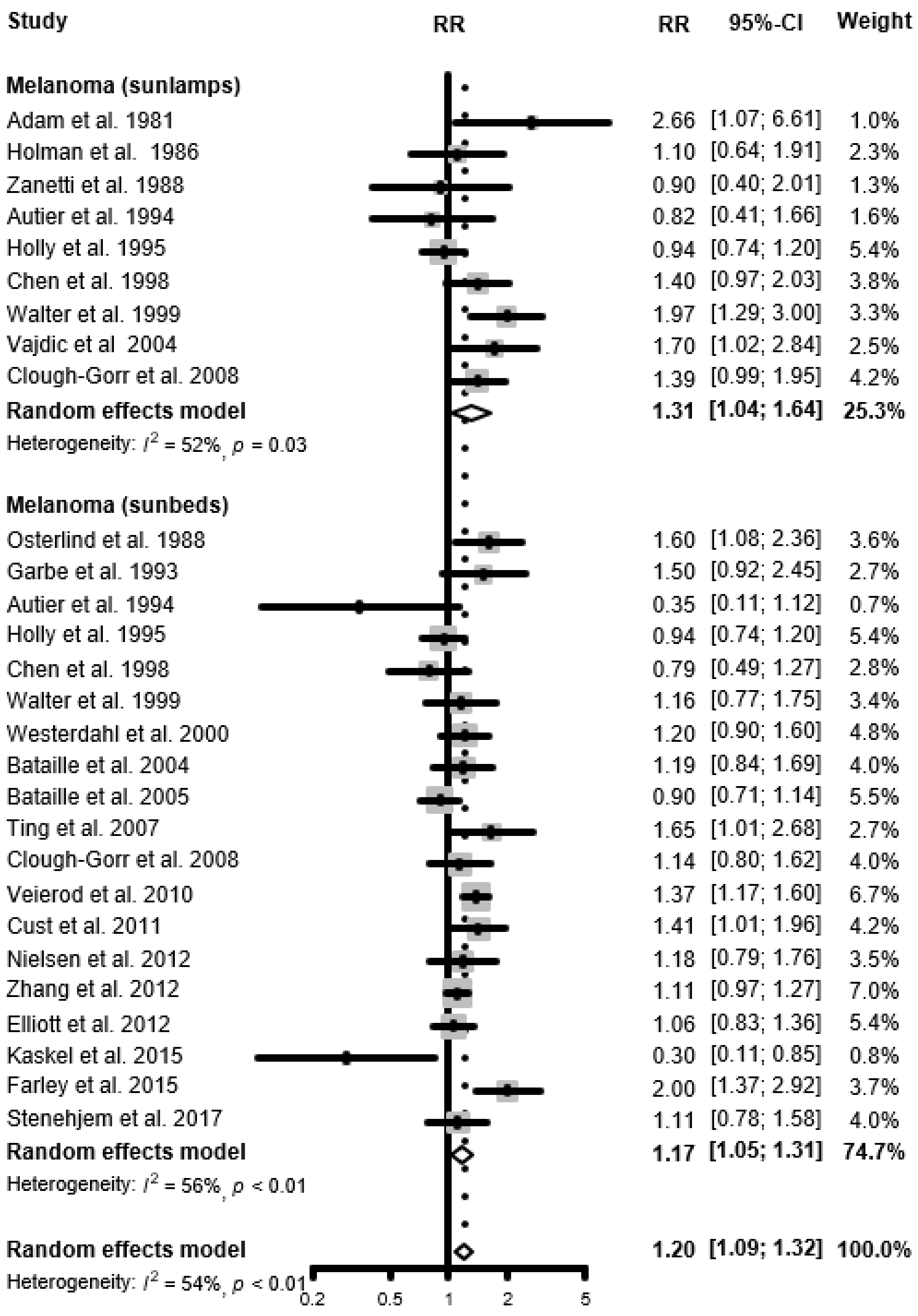

Figure S5. Meta-analysis for risk of melanoma due to different indoor tanning device (sunlamps vs. sunbeds).

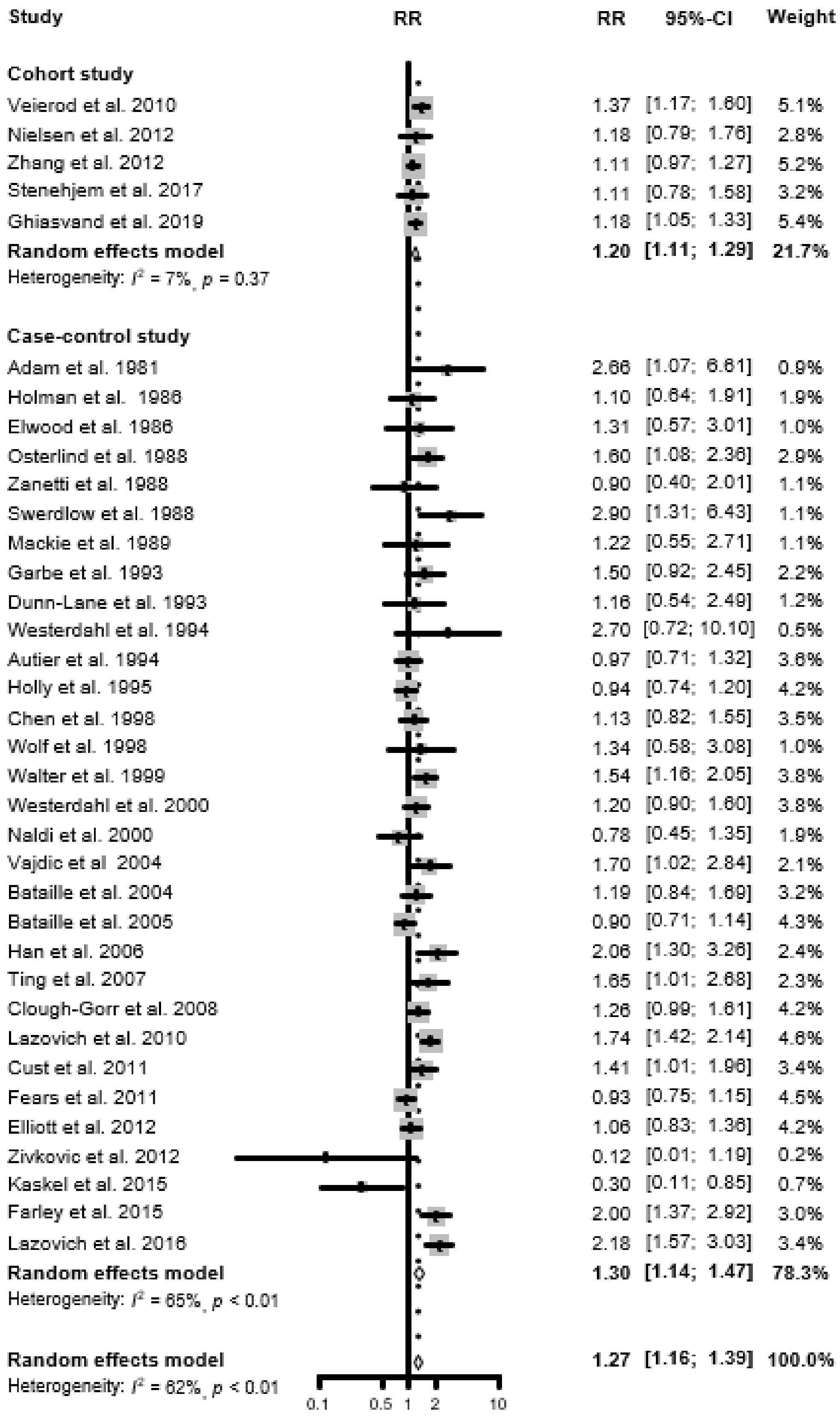
Study
RR
RR 95%-CI Weight
Cohort study
Veierod et al. 2010 1.37 [1.17; 1.60] 5.1%
Nielsen et al. 2012 1.18 [0.79; 1.76] 2.8%
Zhang et al. 2012 1.11 [0.97; 1.27] 5.2%
Stenehjem et al. 2017 1.11 [0.78; 1.58] 3.2%
Ghiasvand et al. 2019 1.18 [1.05; 1.33] 5.4%
Random effects model 1.20 [1.11; 1.29] 21.7%
Heterogeneity: I^2 = 7%, p = 0.37
Case-control study
Adam et al. 1981 2.66 [1.07; 6.61] 0.9%
Holman et al. 1986 1.10 [0.64; 1.91] 1.9%
Elwood et al. 1986 1.31 [0.57; 3.01] 1.0%
Osterlind et al. 1988 1.60 [1.08; 2.36] 2.9%
Zanetti et al. 1988 0.90 [0.40; 2.01] 1.1%
Swerdlow et al. 1988 2.90 [1.31; 6.43] 1.1%
Mackie et al. 1989 1.22 [0.55; 2.71] 1.1%
Garbe et al. 1993 1.50 [0.92; 2.45] 2.2%
Dunn-Lane et al. 1993 1.16 [0.54; 2.49] 1.2%
Westerdahl et al. 1994 2.70 [0.72; 10.10] 0.5%
Autier et al. 1994 0.97 [0.71; 1.32] 3.6%
Holly et al. 1995 0.94 [0.74; 1.20] 4.2%
Chen et al. 1998 1.13 [0.82; 1.55] 3.5%
Wolf et al. 1998 1.34 [0.58; 3.08] 1.0%
Walter et al. 1999 1.54 [1.16; 2.05] 3.8%
Westerdahl et al. 2000 1.20 [0.90; 1.60] 3.8%
Naldi et al. 2000 0.78 [0.45; 1.35] 1.9%
Vajdic et al 2004 1.70 [1.02; 2.84] 2.1%
Bataille et al. 2004 1.19 [0.84; 1.69] 3.2%
Bataille et al. 2005 0.90 [0.71; 1.14] 4.3%
Han et al. 2006 2.06 [1.30; 3.26] 2.4%
Ting et al. 2007 1.65 [1.01; 2.68] 2.3%
Clough-Gorr et al. 2008 1.26 [0.99; 1.61] 4.2%
Lazovich et al. 2010 1.74 [1.42; 2.14] 4.6%
Cust et al. 2011 1.41 [1.01; 1.96] 3.4%
Fears et al. 2011 0.93 [0.75; 1.15] 4.5%
Elliott et al. 2012 1.06 [0.83; 1.36] 4.2%
Zivkovic et al. 2012 0.12 [0.01; 1.19] 0.2%
Kaskel et al. 2015 0.30 [0.11; 0.85] 0.7%
Farley et al. 2015 2.00 [1.37; 2.92] 3.0%
Lazovich et al. 2016 2.18 [1.57; 3.03] 3.4%
Random effects model 1.30 [1.14; 1.47] 78.3%
Heterogeneity: I^2 = 65%, p < 0.01
Random effects model 1.27 [1.16; 1.39] 100.0%
Heterogeneity: I^2 = 62%, p < 0.01
0.1 0.5 1 2 10

Figure S6. Meta-analysis according to study design for risk of melanoma due to indoor tanning device use.

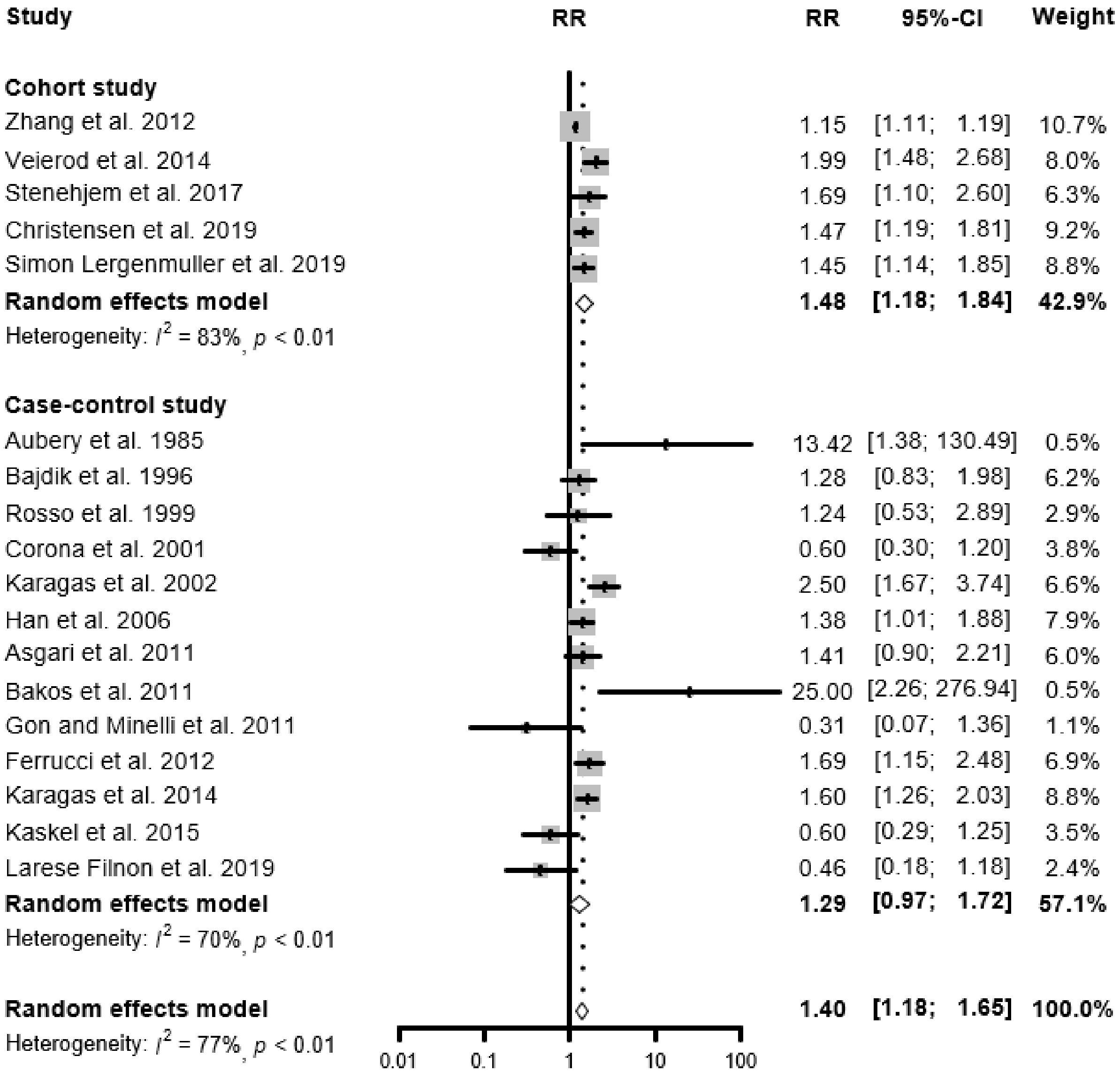

Figure S7. Meta-analysis according to study design for risk of non-melanoma skin cancer (NMSC) due to indoor tanning device use.

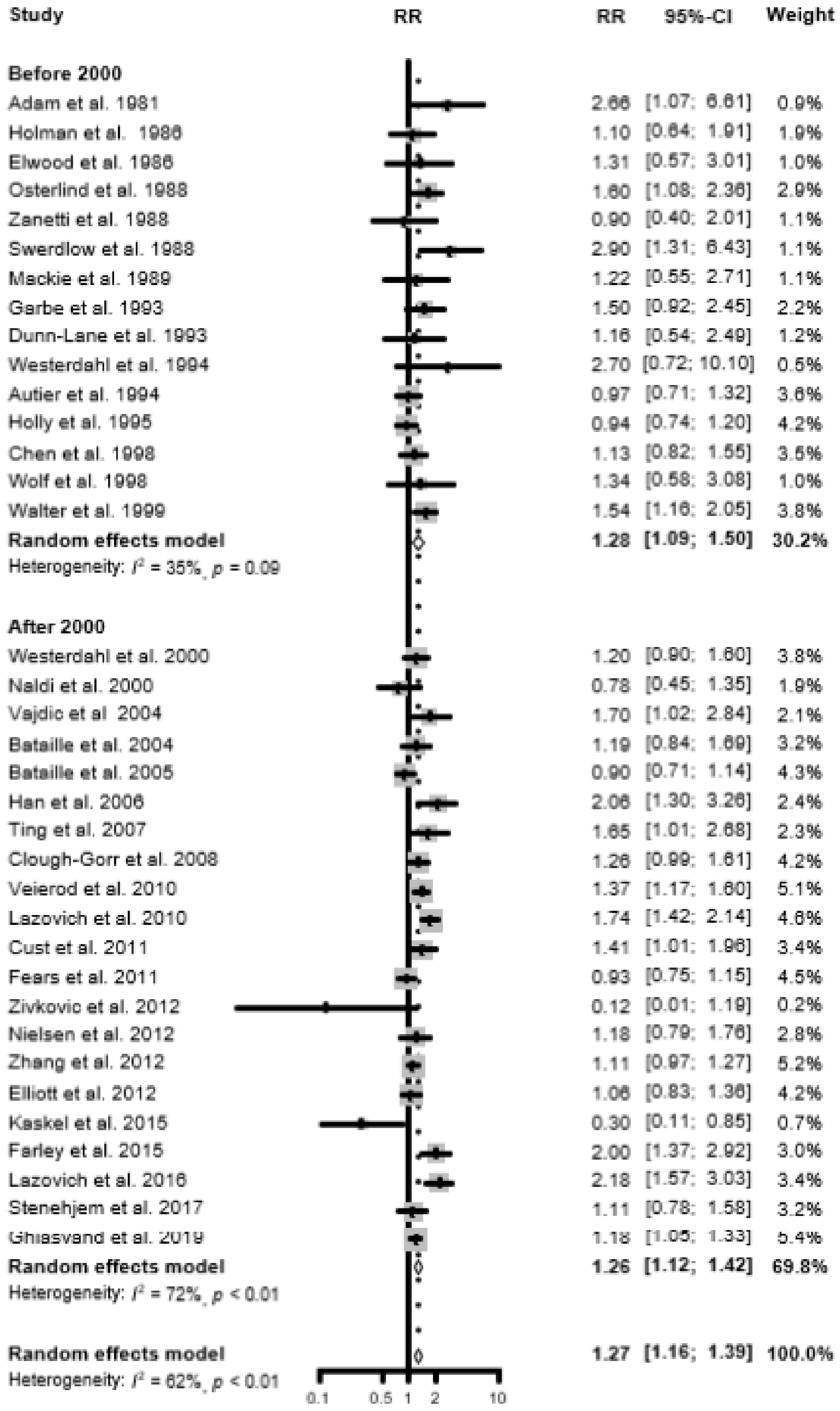
Study
RR
RR 95%-CI Weight
Before 2000
Adam et al. 1981 2.66 [1.07; 6.61] 0.9%
Holman et al. 1986 1.10 [0.64; 1.91] 1.9%
Elwood et al. 1986 1.31 [0.57; 3.01] 1.0%
Osterlind et al. 1988 1.60 [1.08; 2.36] 2.9%
Zanetti et al. 1988 0.90 [0.40; 2.01] 1.1%
Swerdlow et al. 1988 2.90 [1.31; 6.43] 1.1%
Mackie et al. 1989 1.22 [0.55; 2.71] 1.1%
Garbe et al. 1993 1.50 [0.92; 2.45] 2.2%
Dunn-Lane et al. 1993 1.16 [0.54; 2.49] 1.2%
Westerdahl et al. 1994 2.70 [0.72; 10.10] 0.5%
Autier et al. 1994 0.97 [0.71; 1.32] 3.6%
Holly et al. 1995 0.94 [0.74; 1.20] 4.2%
Chen et al. 1998 1.13 [0.82; 1.55] 3.5%
Wolf et al. 1998 1.34 [0.58; 3.08] 1.0%
Walter et al. 1999 1.54 [1.16; 2.05] 3.8%
Random effects model 1.28 [1.09; 1.50] 30.2%
Heterogeneity: I^2 = 35%, p = 0.09
After 2000
Westerdahl et al. 2000 1.20 [0.90; 1.60] 3.8%
Naldi et al. 2000 0.78 [0.45; 1.35] 1.9%
Vajdic et al 2004 1.70 [1.02; 2.84] 2.1%
Bataille et al. 2004 1.19 [0.84; 1.69] 3.2%
Bataille et al. 2005 0.90 [0.71; 1.14] 4.3%
Han et al. 2006 2.06 [1.30; 3.26] 2.4%
Ting et al. 2007 1.65 [1.01; 2.68] 2.3%
Clough-Gorr et al. 2008 1.26 [0.99; 1.61] 4.2%
Veierod et al. 2010 1.37 [1.17; 1.60] 5.1%
Lazovich et al. 2010 1.74 [1.42; 2.14] 4.6%
Cust et al. 2011 1.41 [1.01; 1.96] 3.4%
Fears et al. 2011 0.93 [0.75; 1.15] 4.5%
Zivkovic et al. 2012 0.12 [0.01; 1.19] 0.2%
Nielsen et al. 2012 1.18 [0.79; 1.76] 2.8%
Zhang et al. 2012 1.11 [0.97; 1.27] 5.2%
Elliott et al. 2012 1.06 [0.83; 1.36] 4.2%
Kaskel et al. 2015 0.30 [0.11; 0.85] 0.7%
Farley et al. 2015 2.00 [1.37; 2.92] 3.0%
Lazovich et al. 2016 2.18 [1.57; 3.03] 3.4%
Stenehjem et al. 2017 1.11 [0.78; 1.58] 3.2%
Ghiasvand et al. 2019 1.18 [1.05; 1.33] 5.4%
Random effects model 1.26 [1.12; 1.42] 69.8%
Heterogeneity: I^2 = 72%, p < 0.01
Random effects model 1.27 [1.16; 1.39] 100.0%
Heterogeneity: I^2 = 62%, p < 0.01
0.1 0.5 1 2 10

Figure S8. Meta-analysis according to publication period for risk of melanoma due to indoor tanning device use.

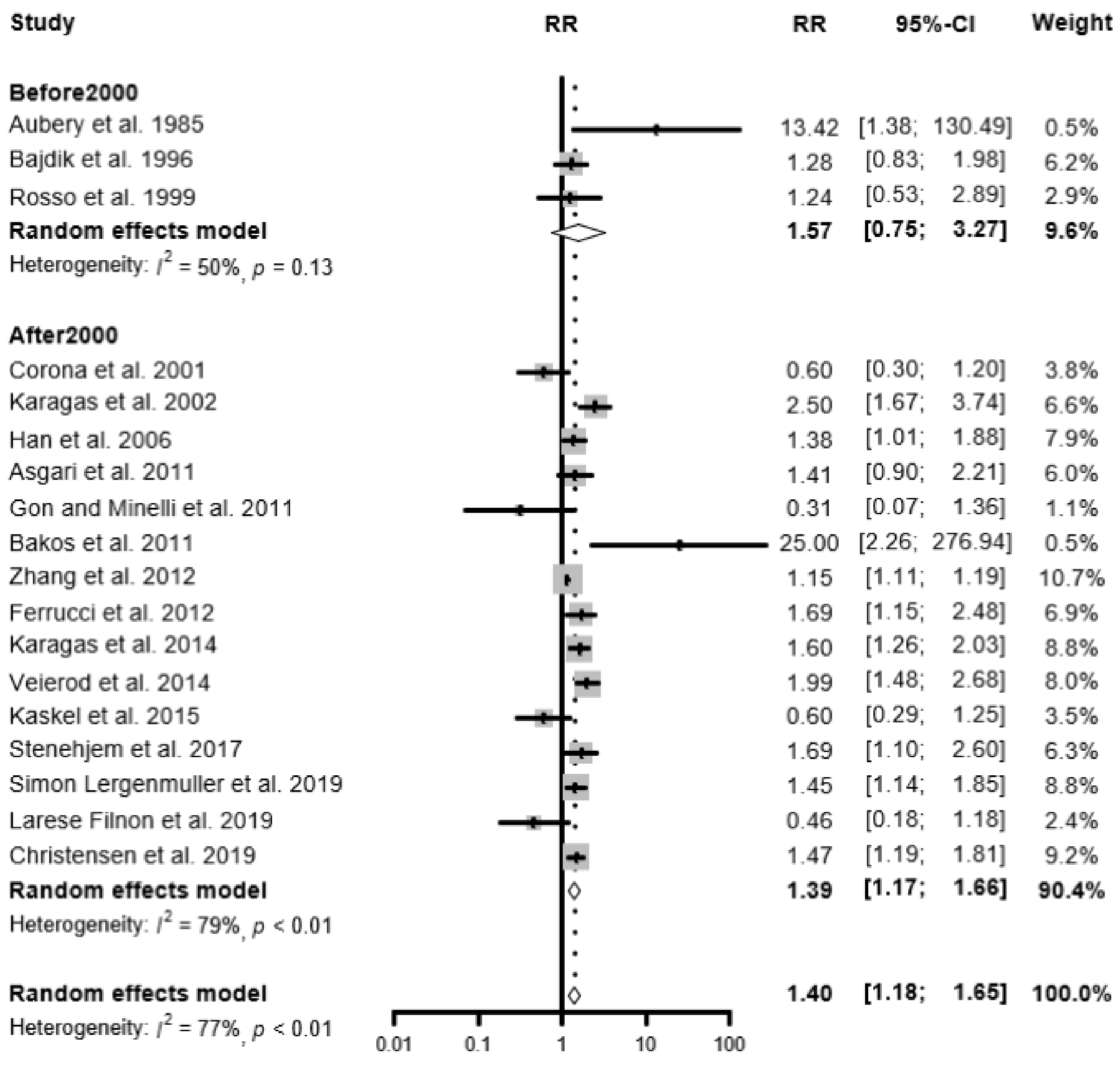

Figure S9. Meta-analysis according to publication period for risk of non-melanoma skin cancer (NMSC) due to indoor tanning device use.

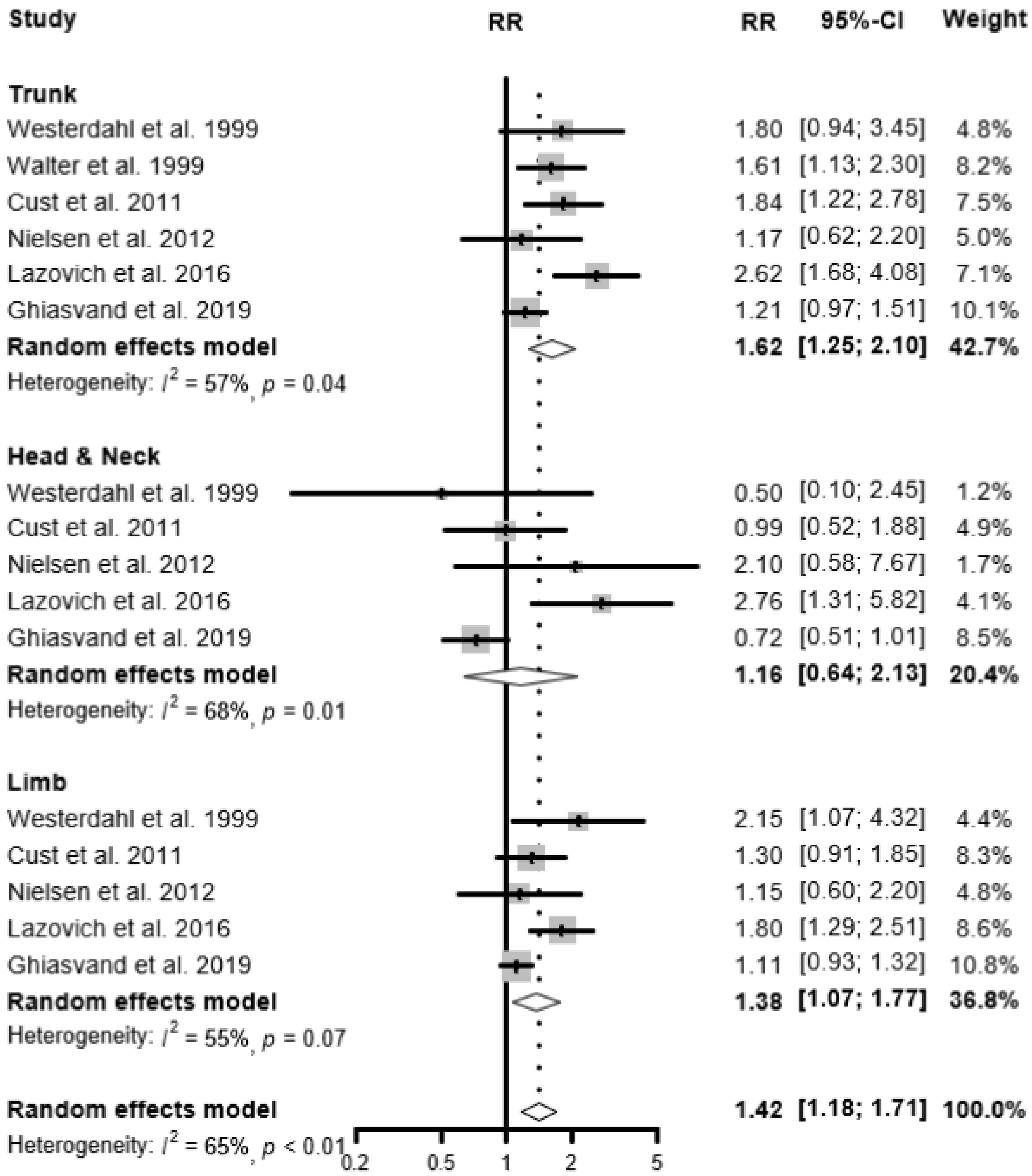

Figure S10. Meta-analysis for risk of melanoma anatomic subtypes due to indoor tanning device use.

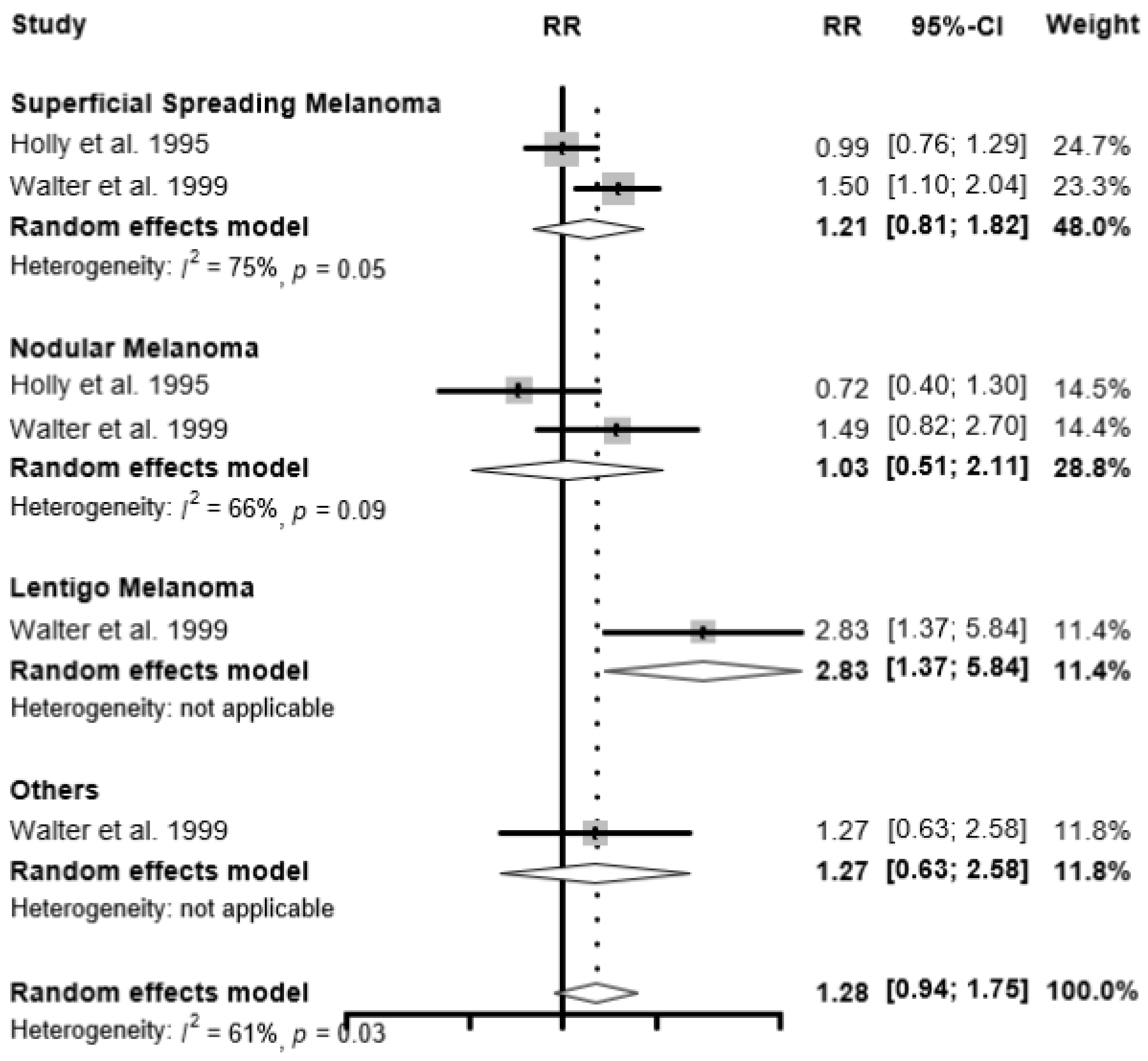

Figure S11. Meta-analysis for risk of melanoma histological subtypes due to indoor tanning device use.

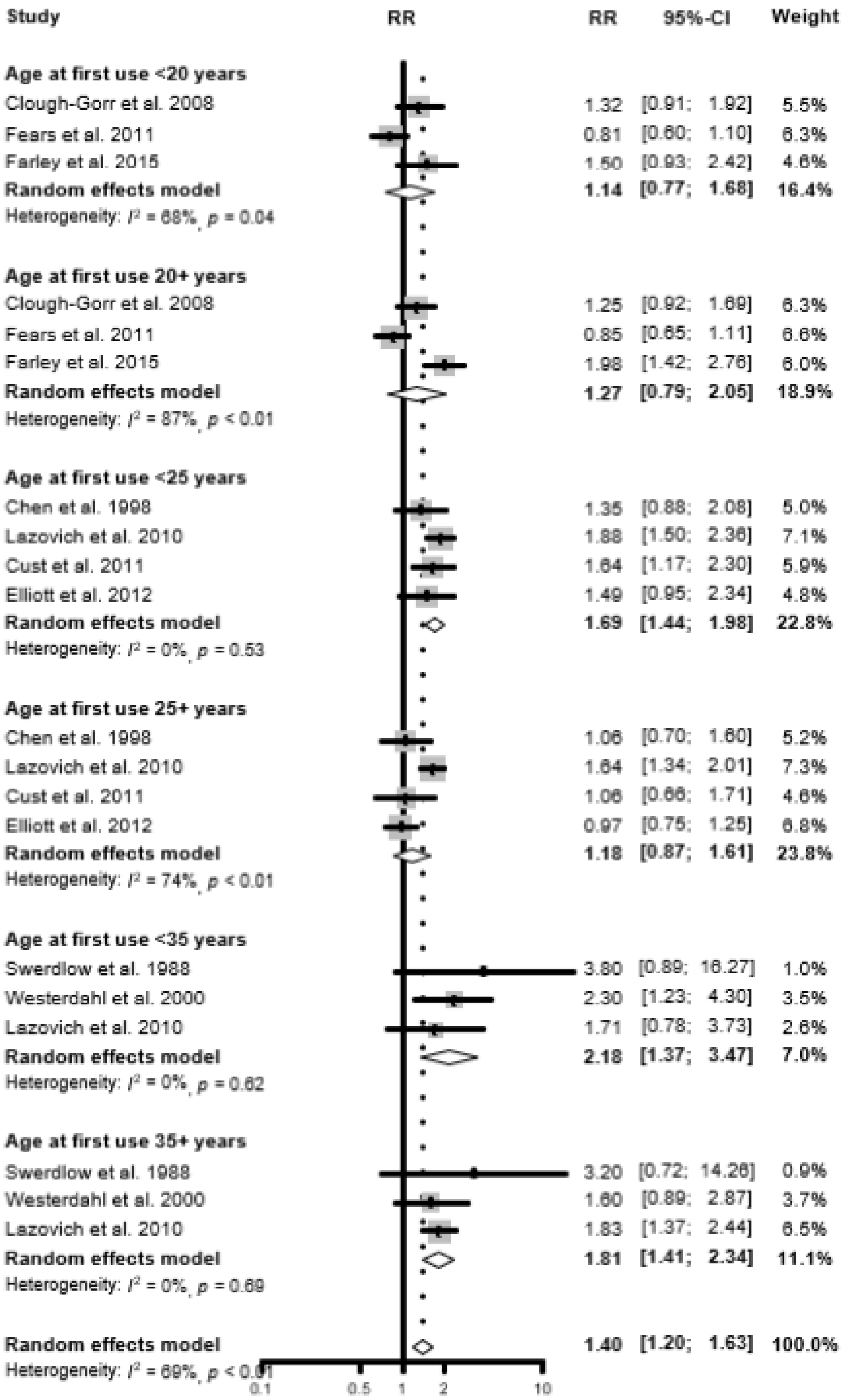

Figure S12. Meta-analysis for risk of melanoma due to age at first indoor tanning device use.

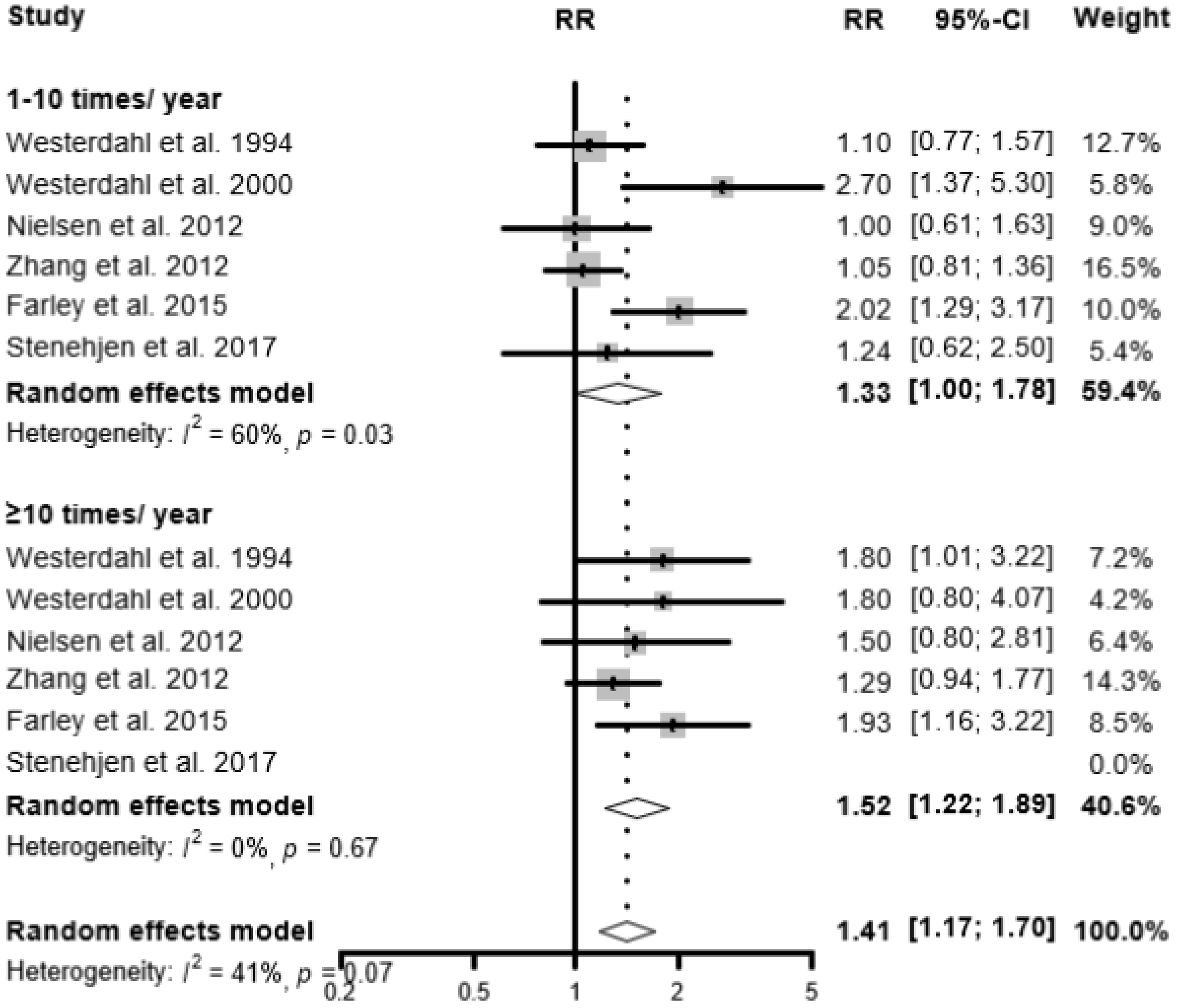

Figure S13. Meta-analysis for risk of melanoma due to the number of indoor tanning device use per year.
